# Effects of Cinacalcet on Post-transplantation Hypercalcemia and Hyperparathyroidism in Adult Kidney Transplant Patients: A Single-Center Experience

**DOI:** 10.7759/cureus.36248

**Published:** 2023-03-16

**Authors:** Nadir Alpay, Alaattin Yıldız

**Affiliations:** 1 Department of Internal Medicine and Nephrology, Faculty of Medicine, Istanbul Aydın University, Istanbul, TUR; 2 Department of Nephrology, Istanbul Faculty of Medicine, Istanbul, TUR

**Keywords:** hypercalcemia, cinacalcet, hyperparathyroidism, secondary hyperparathyroidism, kidney transplantation

## Abstract

Objective: Secondary hyperparathyroidism may manifest as hypercalcemia in the post-transplant period. The classical treatment method is parathyroidectomy and the alternative is oral cinacalcet, a calcimimetic agent therapy. We retrospectively investigated the effect of cinacalcet therapy on kidney and patient survival in these patients.

Materials and methods: In our single-center, retrospective, observational study, files of 934 patients who underwent renal transplantation in our unit between 2008 and 2022 were reviewed. A total of 23 patients were started on cinacalcet for the treatment of hypercalcemia (calcium > 10.3 mg/dl) and parathyroid hormone (PTH) elevation (>65 pg/ml). Patients with calcium < 10.3 mg/dl and PTH > 700 pg/ml at any time in the follow-up after renal transplantation were included in the study. In addition, the demographic data of the patients, baseline creatine, calcium, phosphorus, and PTH levels at the time of hypercalcemia, parathyroid ultrasonography, parathyroid scintigraphy, creatinine, calcium, phosphorus, and PTH levels in the last controls, and survival status were evaluated.

Results: The mean age of 23 patients included in the study was 52.7 ± 11 years (minimum: 32; maximum: 66). Of the patients, 16 (69.6%) were male, and 15 (65.2%) were transplanted from a living donor. Parathyroid scintigraphic revealed adenoma in three (13%) patients, hyperplasia in five patients (21.7%), and no involvement in 15 patients (65.2%). Cinacalcet treatment was initiated at a median of 33 months (interquartile range (IQR) = 13-96) after the kidney transplant operation. There was no graft loss in the patients during the follow-up period. Twenty-two patients (95.7%) were alive, and one patient died. The calcium level of the patients decreased from 11.3 ± 0.64 mg/dl to 9.98 ± 0.78 mg/dl (p = 0.001) after cinacalcet treatment. Phosphorus values increased from 2.7 ± 0.65 mg/dl to 3.10 ± 0.65 mg/dl (p = 0.004). On the other hand, there was no significant difference in PTH levels between the initial and final controls (285 (IQR = 150-573) vs. 260 pg/ml (IQR = 175-411), p = 0.650). Also, creatinine levels were similar (1.2 ± 0.38 vs. 1.24 ± 0.48 mg/dl, p = 0.43). Despite cinacalcet treatment, calcium levels did not decrease in eight patients. Complications such as renal dysfunction and pathological fracture did not develop in these patients.

Conclusions: It seems that cinacalcet treatment is a suitable option for patients with hypercalcemia and/or hyperparathyroidism with low drug interactions and good biochemical control after renal transplantation.

## Introduction

Renal transplantation is the most effective treatment for end-stage renal disease and is also an effective treatment for metabolic abnormalities of chronic kidney disease. However, it can only partially correct mineral and bone disorders. Secondary hyperparathyroidism seen in varying degrees during the dialysis period may manifest as hypercalcemia in the post-transplant period. Apart from its bone metabolism and cardiovascular effects, hypercalcemia is an electrolyte disorder that can also damage the transplanted kidney and should be treated. The classical treatment method is parathyroidectomy, and the alternative is oral cinacalcet therapy.

Considering the potential harms of surgical procedures in the post-transplant period, alternative treatment approaches have come to the fore for hypercalcemia and secondary hyperparathyroidism. Oral cinacalcet therapy suppresses parathyroid hormone (PTH) secretion with a calcium-sensitive receptor-like effect in the parathyroid gland. Secondary hyperparathyroidism after renal transplantation has been reported in 10% to 66% [1,2]. This difference in rate is due to the difference in PTH level and measurement time for diagnosis. High and prolonged PTH stimulation due to low calcium and high phosphorus during dialysis may lead to parathyroid gland hyperplasia or autonomous hormone-producing adenomas. This situation constitutes a large part of secondary hyperparathyroidism and hypercalcemia in the post-transplant period [3,4].

Although normal renal function and 1,25 dihydroxy vitamin D3 levels return to normal after transplantation, PTH levels generally remain elevated. Thus, it increases serum calcium levels and urinary phosphate excretion and decreases phosphorus in the blood, which can also cause a decrease in bone mass. The most important predictive risk factors for the post-transplant development of secondary hyperparathyroidism are blood calcium, alkaline phosphatase, and PTH levels at the time of transplantation, type of dialysis, and graft function [4,5]. Low 25-hydroxy (OH) vitamin D levels post-transplant also contribute to the situation [6].

Although cardiovascular mortality in renal transplant patients is lower than in the dialysis patient population, it is higher than in the normal population [7,8]. In addition to traditional risk factors such as hypertension, diabetes, and hyperlipidemia, lifelong immunosuppressive drugs (tacrolimus, cyclosporine, everolimus, and corticosteroids) use and their complications are also added to risk factors in these patients [9]. Receptors for PTH have been identified in cardiomyocytes, endothelial, and vascular smooth muscle cells. These receptors identified for PTH suggest that PTH has possible effects in the defined tissues apart from its effects on bones and kidneys [10].

The decrease in serum calcium level and PTH level with cinacalcet treatment has been shown previously in cases of primary hyperparathyroidism or secondary hyperparathyroidism in dialysis patients [11,12]. Recently, it is being used off-label in renal transplant patients because of the lack of consent. We also screened hypercalcemia and elevated parathormone in patients with kidney transplantation followed in our unit. We retrospectively investigated the effect of cinacalcet therapy, a calcimimetic agent, on kidney and patient survival in these patients.

## Materials and methods

We screened the records of 934 patients who had undergone renal transplantation at our hospital between 2008 and 2022. A total of 23 patients were started on cinacalcet for the treatment of hypercalcemia (calcium > 10.3 mg/dl) and PTH elevation (>65 pg/ml). Patients with calcium < 10.3 mg/dl and PTH > 700 pg/ml at any time in the follow-up after renal transplantation were included in the study. Patients who followed for at least six months were included. Patients with hypercalcemia and elevated PTH levels other than secondary hyperparathyroidism (e.g. malignancies and bone metastases) were excluded. Patients using drugs that may cause hypercalcemia (such as calcium and vitamin D) were excluded from the study. Calcium levels of 8.5-10.3 mg/dl, phosphorus levels of 2.5-4.5 mg, and PTH levels of 15-65 pg/ml were accepted as normal values. Those patients in whom hypercalcemia (>10.3) did not develop even if the PTH level was >65 pg/ml and who were not given cinacalcet treatment were not included. Even if hypercalcemia did not develop, patients with PTH > 700 pg/ml who were treated with cinacalcet were included.

Oral cinacalcet therapy was started at 30 mg/day in selected patients and increased to 60 mg according to the patient's tolerance in the follow-up. Along with the demographic data of the patients, duration of hemodialysis, etiologies of primary kidney disease, dates, number, and types of renal transplantation, immunosuppressive drugs, baseline creatine, calcium, phosphorus, and PTH levels at the time of hypercalcemia, parathyroid ultrasonography (USG) and scintigraphy, creatinine, calcium, phosphorus, and PTH levels in the last controls, and survival status were evaluated.

The patients participating in the study were given detailed information about the purpose and content of the study and their consent was obtained. Our study was approved by Istanbul Aydın University Non-Invasive Clinical Research Ethics Committee and was directed in accordance with the Declaration of Helsinki (decision no: B.30.2.AYD.0.00.00-050.06.04/19).

## Results

The mean age of 23 patients included in the study was 52.7 ± 11 years (minimum: 32; maximum: 66). Of the patients, 16 (69.6%) were male and seven (30.4%) were female (Table 1). The mean age of transplantation of the patients was 47.5 ± 12.5 years (minimum: 22; maximum: 65). When primary kidney diseases of the patients were evaluated, three (13%) had amyloidosis, three (13%) had hypertensive nephropathy, two (8.7%) had vesicourethral reflux (VUR), 12 (52%) had unknown etiology, and one patient had focal segmental glomerulosclerosis (FSGS), multicystic kidney, and urethral stricture. Of the patients, two (8.7%) had second transplantation, 15 (65.2%) were transplanted from a living donor, and eight (34.8%) had cadaveric transplantation. Two patients had undergone parathyroidectomy surgery before transplantation.

**Table 1:** Baseline characters at the start of cinacalcet treatment CYC: cyclosporine; AZA: azathioprine; TAC: tacrolimus; MPM: mycophenolate mofetil; MPS: mycophenolate sodium; PRD: prednisolone; SD: standard deviation; IQR: interquartile range.

Features	Results
Age (mean ± SD)	52.7 ± 11
Gender: Male	16
Female	7
Renal transplantation, age (mean ± SD)	47.5 ± 12.5
Follow-up time, months (mean ± SD)	14.6 ± 7.4
Hemodialysis times, years (median (IQR))	7 (1-10)
Retransplantation number	2
Living donor	15
Cadaveric donor	8
Parathyroidectomy operation history	2
Primary kidney diseases	
Amyloidosis	3
Hypertensive nephropathy	3
Vesicourethral reflux (VUR)	2
Focal segmental glomerulosclerosis (FSGS)	1
Urethra stenosis	1
Multicystic kidney	1
Etiology unknown	12
Parathyroid ultrasonography: Nodule	11
No nodule	12
Parathyroid scintigraphy: Adenoma	3
Hyperplasia	5
None	15
Post-transplant cinacalcet initiation, months (median (IQR))	33 (13-96)
Immunosuppressives	
Group 1: TAC + MPM/MPS + PRD	17
Group 2: TAC + AZA + PRD	4
Group 3: CYC + MPM/MPS + PRD	2
Cinacalcet initial laboratory values	
Calcium, mg/dl (mean ± SD)	11.3 ± 0.64
Phosphorus, mg/dl (mean ± SD)	2.7 ± 0.65
Creatinine, mg/dl (mean ± SD)	1.2 ± 0.38
Parathyroid hormone (PTH), pg/ml (mean ± SD)	285 (IQR = 150-573)
Patient survival: Alive	22
Dead	1

The median duration of hemodialysis was seven years (interquartile range (IQR) = 1-10). At the beginning of cinacalcet treatment, calcium was 11.3 ± 0.64 mg/dl, phosphorus was 2.7 ± 0.65 mg/dl, creatinine was 1.2 ± 0.38 mg/dl, and PTH was 285 pg/ml (IQR = 150-573). Immunosuppressive drugs used in group 1 were tacrolimus (TAC) + mycophenolate mofetil (MPM)/mycophenolate sodium (MPS) + prednisolone (PRD) in 17 patients (73.9%); group 2: TAC + azathioprine (AZA) + PRD in four patients (17.4%); group 3: cyclosporine (CYC) + MPM/MPS + PRD in two patients (8.7%). While 11 (47.8%) patients had nodules on parathyroid ultrasonography, no nodules were detected in 12 (52.2%) patients. Parathyroid scintigraphic examination revealed no adenoma in three (13%) patients, hyperplasia in five patients (21.7%), and no involvement in 15 patients (65.2%). The initiation of cinacalcet treatment was initiated at a median of 33 (IQR = 13-96) months after the renal transplant operation. After cinacalcet treatment, the patients were followed up for an average of 14.6 ± 7.4 months. Calcium was 9.9 ± 0.78 mg/dl, phosphorus was 3.1 ± 0.67 mg/dl, creatinine was 1.24 ± 0.48 mg/dl, and final PTH was 260 pg/ml (IQR = 125-411) in the final controls after follow-up.

There was no graft loss in the patients during the follow-up period. Twenty-two patients (95.7%) were alive, and one patient (heart valve replacement) with a ruptured mitral valve with acute heart failure using Coumadin died. The calcium level of the patients decreased from 11.3 ± 0.64 mg/dl to 9.98 ± 0.78 mg/dl after cinacalcet treatment (p = 0.001). Phosphorus values increased from 2.7 ± 0.65 mg/dl to 3.10 ± 0.65 mg/dl (p = 0.004). On the other hand, there was no significant difference in parathormone levels between the initial and final controls: PTH = 260 pg/ml (IQR = 175-411; p = 0.650). Also, creatinine levels were similar at 1.24 ± 0.48 mg/dl (p = 0.43) (Table 2). None of the patients' treatment was discontinued due to a side effect during the cinacalcet treatment but the calcium level did not decrease in eight patients despite the treatment calcium > 10.3. Complications such as renal dysfunction and pathological fractures did not develop in these patients.

**Table 2:** Comparative values of baseline and final status after cinacalcet treatment Statistically significant values are in bold (p < 0.05).

Baseline values at the beginning of cinacalcet	Cinacalcet post-treatment values	P-value
Calcium (mg/dl): 11.3 ± 0.64	Calcium (mg/dl): 9.9 ± 0.78	0.001
Phosphorus (mg/dl): 2.7 ± 0.65	Phosphorus (mg/dl): 3.1 ± 0.67	0.004
Creatinine (mg/dl): 1.2 ± 0.38	Creatinine (mg/dl): 1.24 ± 0.48	0.435
Parathormone (pg/ml): 285 (IQR = 150-573)	Parathormone (pg/ml): 260 (IQR = 125-411)	0.650

## Discussion

We retrospectively screened patients with hyperparathyroidism who received cinacalcet therapy due to hypercalcemia in the post-transplant period and we evaluated their renal and patient survivals. Although there was no significant decrease in PTH levels after an average of 14 months of follow-up, we observed a significant decrease in calcium levels and a significant increase in phosphorus levels (Table 2). In one of the first studies in this field, Kruse et al. (2005) studied 14 stable renal transplant patients. While PTH levels did not change, a decrease in calcium and an increase in phosphorus were detected in the three-month follow-up. It was tested only in two patients with very variable PTH levels, two hours after taking cinacalcet; PTH decreased to 80-50 pg/ml but increased again to the pre-drug value in the measurement eight hours after the drug. In addition, creatinine levels remained stable in our study contrary to a slight increase in this study [13].

Çeltik et al. showed that if hypercalcemia is not treated, it causes nephrocalcinosis and tubulointerstitial calcification. Here, 247 post-transplant patients with hypercalcemia were followed for six and 12 months, and a protocol biopsy was performed. Tubulointerstitial calcification was found in the biopsy in 70-90% of the patients [14]. In a study by Serra et al. in the 10-week follow-up of 11 renal transplant patients, calcium levels decreased in all patients, phosphorus levels increased, and renal functions remained stable. In fact, a 21.8% decrease in PTH levels compared to baseline was also detected by the authors [15].

In a study by Rivelli et al. in which cinacalcet treatment was followed for three years in 46 patients, it was shown that the calcium level returned to normal and the PTH levels decreased significantly after three years (317 ± 242 vs. 145 ± 72 pg/ml), and it was stated that only one patient had resistant hypercalcemia and was given a parathyroidectomy operation. In addition, it was observed that cinacalcet was well tolerated for three years with the most common side effect being gastrointestinal symptoms [16]. Zavvos et al. also conducted a study including the results of a five-year follow-up with cinacalcet treatment. They also showed that calcium decreased significantly, phosphorus increased, PTH levels decreased over time, and renal functions were preserved [17].

It is known that cinacalcet decreases calcium absorption from the kidney proximal tubules by lowering PTH and increases urinary calcium excretion. Courbebaisse et al. reported that long-term use of cinacalcet caused renal damage in a randomized study including 71 renal transplant patients with hypercalcemia and hyperparathyroidism. Renal biopsy was performed before and after three and 12 months of follow-up. Although urinary calcium excretion was two times higher in the cinacalcet group, no negative effect was found on calcium deposits and renal functions in the biopsy results [18].

Evenepoel et al. published three-year prospective follow-up results of 21 patients who used cinacalcet in the pre-transplant period and discontinued during renal transplantation and 303 renal transplant patients who never used cinacalcet before transplant. In this study, there was no difference in mineral metabolism and renal functions between the patients who used cinacalcet and those who did not use it in the third and 12th months. Moreover, renal biopsy results showed 45% more nephrocalcinosis in cinacalcet users, and the rate of parathyroidectomy in the follow-up was 28.6% in the cinacalcet group versus 7.2% in the not users group. It was concluded that using cinacalcet did not affect the course of secondary hyperparathyroidism in patients awaiting or receiving kidney transplantation and that rebound hyperparathyroidism and high parathyroidectomy rates were encountered when the drug was discontinued after transplantation [19].

Paschoalin et al. also followed 41 patients who were started on cinacalcet due to post-transplant hypercalcemia. Fourteen patients were discontinued at the end of one year. In the follow-up, 50% of these discontinued patients were restarted on cinacalcet due to hypercalcemia. It is stated here that it is unknown how much the drug will be used and when it will be discontinued [20]. Cruzado et al. compared cinacalcet with parathyroidectomy in 15 patients in each group who were followed up for one year in a randomized controlled prospective study. At the end of one year, 10 out of 15 patients achieved normocalcemia in the cinacalcet group, while 15 out of 15 patients in the parathyroidectomy group achieved normocalcemia. Phosphate levels returned to normal in all patients in both groups. Renal functions were preserved in both groups, there was no significant difference between proteinuria levels, and vascular calcification scores were similar. PTH levels decreased significantly more in the parathyroidectomy group and the bone mineral density of the femoral neck was significantly increased. According to the cost analysis, it was stated that parathyroidectomy was more effective if the cinacalcet treatment was continued for 14 months. With all these results, they emphasized that subtotal parathyroidectomy is superior to cinacalcet [21].

Cohen et al. conducted a meta-analysis of all studies using cinacalcet for post-transplant hypercalcemia and hyperparathyroidism between 2004 and 2012. In total, 21 studies with 411 kidney transplant patients were included. The patients were followed for three to 24 months. Calcium decreased by 1.14 mg/dl, phosphorus increased by 0.46 mg/dl, and PTH decreased by 102 pg/ml. There was a decrease of 0.02 mg/dl in serum creatinine, but no significant change was observed. The most common side effect was gastrointestinal intolerance. Seven patients from five studies treated with cinacalcet developed hypocalcemia. Long follow-up periods and clinical results with randomized studies were recommended to confirm these findings for showing the safety of cinacalcet after transplantation [22].

The limitations of our study are that it was a single-center experience with a short follow-up period and a low number of patients. Moreover, additional information such as 25 OH vitamin D, fibroblast growth factor-23 (FGF-23) level, dual-energy X-ray absorptiometry (DEXA) bone density, and vascular calcification scores, which were checked in some other studies but not ours, would be very useful in evaluating the patients.

## Conclusions

As a result, in the post-transplant period, secondary hyperparathyroidism of varying degrees remaining from the dialysis periods of the patients resolves spontaneously in most patients with the improvement of the uremic picture and close biochemical follow-up. Some patients require treatment because they have autonomy parathyroid tissue, hypercalcemia, high PTH, and cardiovascular and bone mineral metabolism disorders. Although parathyroidectomy treatment provides better biochemical and metabolic control, it is necessary to consider the surgical risk, complications, and lack of experienced surgeons in kidney transplant patients. Although oral cinacalcet therapy is not approved in this indication, it seems to be a suitable option for patients with a renal transplant who have low drug interactions, good biochemical control, and a high risk of surgical operation. It would be a rational approach to decide on the treatment by considering the pros and cons of both treatment methods and following the results closely.
